# Geometric validation of a computer simulator used in radiography education

**DOI:** 10.1259/bjro.20190027

**Published:** 2020-02-03

**Authors:** Philip Cosson, Zenghai Lu

**Affiliations:** 1 Teesside University, Middlesbrough, United Kingdom

## Abstract

**Objectives:**

The radiographical process of projection of a complex human form onto a two-dimensional image plane gives rise to distortions and magnifications. It is important that any simulation used for educational purposes should correctly reproduce these. Images generated using a commercially available computer simulation widely used in radiography education (ProjectionVR^TM^) were tested for geometric accuracy of projection in all planes.

**Methods:**

An anthropomorphic skull phantom was imaged using standard projection radiography techniques and also scanned using axial CT acquisition. The data from the CT was then loaded into the simulator and the same projection radiography techniques simulated. Bony points were identified on both the real radiographs and the digitally reconstructed radiographs (DRRs). Measurements sensitive to rotation and magnification were chosen to check for rotation and distortion errors.

**Results:**

The real radiographs and the DRRs were compared by four experienced observers and measurements taken between the identified bony points on each of the images obtained. Analysis of the mean observations shows that the measurement from the DRR matches the real radiograph +1.5 mm/−1.5 mm. The Bland Altman bias was 0.55 (1.26 STD), with 95% limits of agreement 3.01 to −1.91.

**Conclusions:**

Agreement between the empirical measurements is within the reported error of cephalometric analysis in all three anatomical planes. The image appearances of both the real radiographs and DRRs compared favourably.

**Advances in knowledge:**

The commercial computer simulator under test (ProjectionVR^TM^) was able to faithfully recreate the image appearances of real radiography techniques, including magnification and distortion. Students using this simulation for training will obtain feedback likely to be useful when lessons are applied to real-world situations.

## Introduction

The ability to ‘produce accurate and correct images’ is seen by qualified radiographers as one of the most important competencies. Andersson et al. (2012) surveyed 406 qualified staff of all levels of experience. This competence was scored as the sixth priority out of the 29 competencies evaluated.^1^ In the same study, the accurate projection and collimation of an image was seen as the most important technical competency by radiographers with less than 5 years’ experience. Gaining this competence is not trivial; more than 200 unique diagnostic radiographic projections are in standard use.^2^ It is an important area of training, as having to repeat a radiographic exposure is a major cause of increased risk to patients in projection radiography.^3^ Findings of major studies suggest that it is errors in patient positioning, centring and collimation that are the main reasons radiographic examinations are repeated.^4–6^ These repeats are not only implicated in increased risks, but also patient dissatisfaction, reduction in capacity and increased waiting times. The most frequently occurring repeated examination types are shoulder, hip, spines, in-department chest, skull/facial bones and pelvis.^7^

As there is no way of eliminating the potential for harm due to projection radiography, students cannot expose patients to ionising radiation without direct supervision from a qualified person. Supervisors will never allow students to make foreseeable mistakes.^8–10^ Mason (2006) surveyed 82 student radiographers and found that students felt there was often ‘too much supervision’. In the opinion of the students, these supervisors often 'unnecessarily step in’ to make changes to the patient position or examination settings before exposure ‘to ensure patient safety’.^11^ Necessary supervision could hinder student development of their own practice via reflection.

In radiography and other healthcare practice where the public and/or the trainee may come to harm, computer simulation is now available as a cost-effective solution.^12–14^ A validated computer simulation has the potential to enhance student experience by providing opportunities for reflection without an ethical burden, high costs or legislative compliance issues. Furthermore, students have been used to technological assistance with their learning for many years and desire the ability to study at their own pace in their own time.^15^

**Figure 1.**
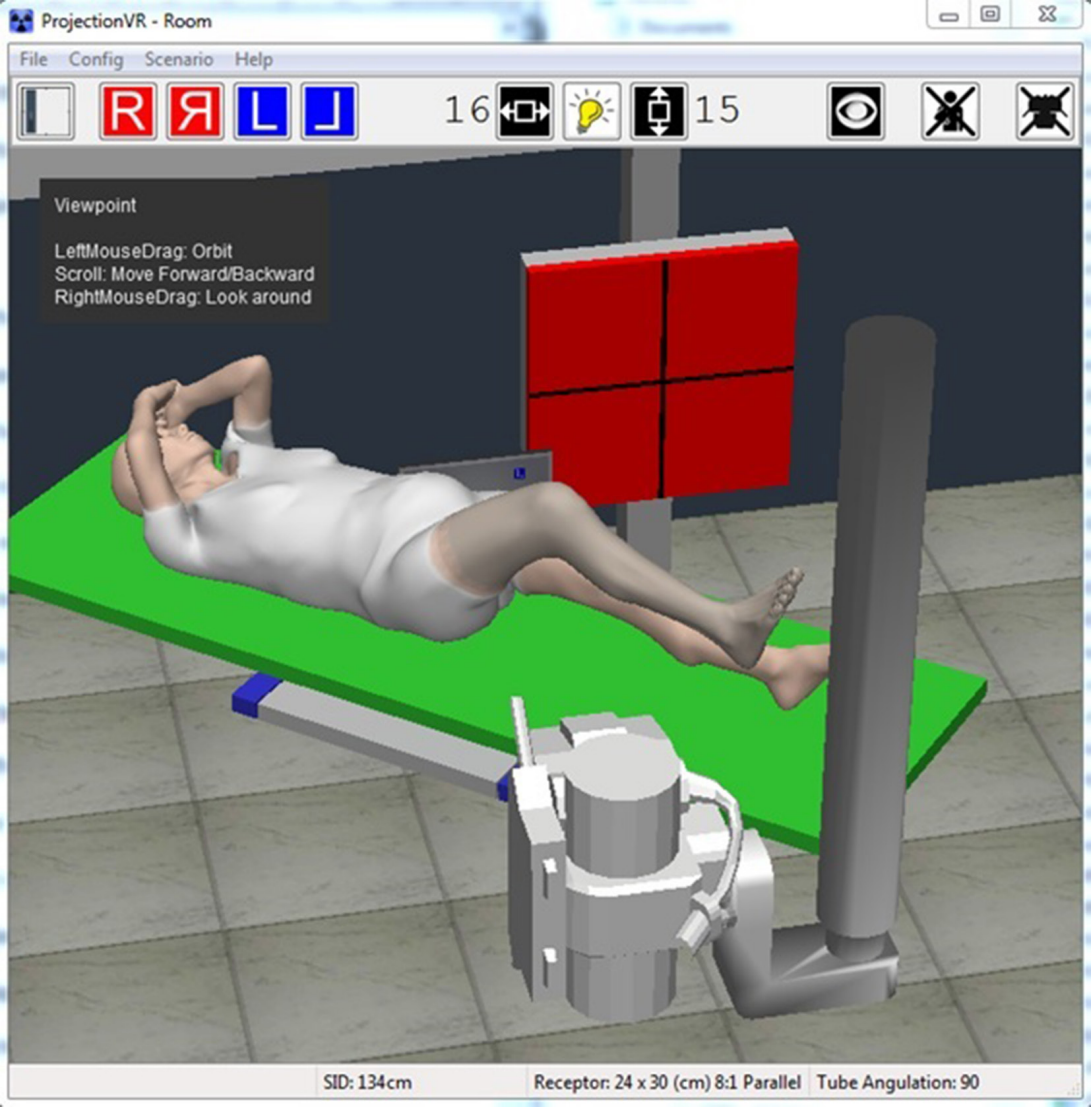
ProjectionVR^TM^ (v. 5.0) X-ray room interface.

**Figure 2.**
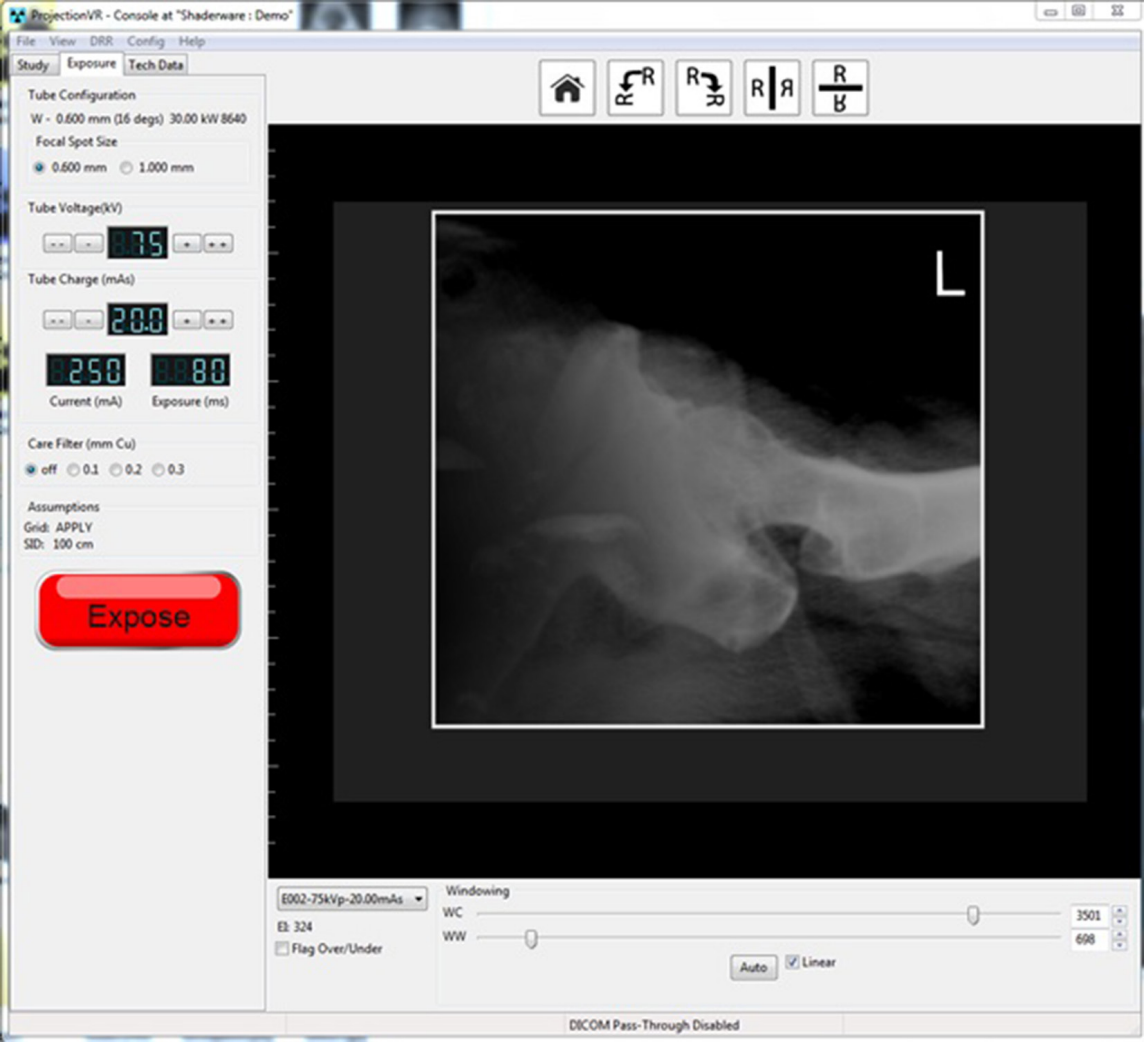
ProjectionVR^TM^ (v. 5.0) X-ray console displaying digitally reconstructed radiograph (DRR).

Since 2006, a computer simulation of a diagnostic radiography environment has been commercially available (ProjectionVR^TM^, Shaderware Limited).^16^ At the time of writing, this has been widely adopted, by over 160 universities and colleges in 24 countries.^17^ This simulation provides a ‘window’ into a virtual environment consisting of the usual features of an X-ray room (Figure 1). The simulated X-ray tube, patient and receptor can be positioned to obtain virtual radiographic projections. The resultant simulated, synthetic or ‘virtual radiograph’ is then calculated using a computer algorithm and displayed to give students feedback (Figure 2).

With any simulator, there is a risk of negative training effect.^18^ The first requirement must be the accurate generation of the ‘virtual radiograph’, as this is the main feedback the student will receive. This work aims to validate geometrically the algorithm underlying this widely used commercial diagnostic radiography simulation (ProjectionVR^TM^, Shaderware). This algorithm will be empirically tested, by comparing the rendering of 3D geometry (a phantom) onto a 2D receptor (the image plane), with that of a real X-ray beam, phantom and receptor system. The results will enable judgements concerning the validity of the resultant ‘virtual radiographs’ from this algorithm, and therefore the accuracy of feedback to the student users.

## Literature review

Students are traditionally introduced to the terminology and practice of position and projection in clinical skills labs at Higher Education Institutions (HEIs). Rosenkoetter (2007) surveyed radiography program directors in the USA and reported that practical lab-based tuition is increasingly seen as essential.^19^ Remmen et al. (1998,1999) believed that clinical practice alone in medical training was unable to deliver adequate clinical skills training.^20,21^ Indeed, in modern healthcare education, it has been stated by influential researchers and health regulators that...

‘There is growing pressure for the training process to be transparent, for it to be underpinned by objective measures of skill, and for alternatives to patient-based training to be used wherever possible’.^22^

‘[Healthcare professionals] should learn skills in a simulation environment and using other technologies before undertaking them in supervised clinical practice’.^23^

There are those who go further, and consider it unethical to allow practice with a patient without first training on simulators.^24^ Professional bodies have advocated the use of simulation,^25^ some allowing a percentage of mandatory clinical hours to be ‘simulated practice.’^26^ Such is the demand for a lifelike simulation of radiography practice that even cadavers have been proposed for positioning training.^27^ Solutions to the limitations on student clinical experience have been sought despite constraints such as access to human remains, and the high associated costs of the radiographic equipment, phantoms and necessary small group lab teaching.^28^

There has been considerable research interest in simulation in the diagnostic radiography literature.^29–34^ However, no attempt at geometric validation of a general radiography simulation has been published. ‘Virtual radiographs’, commonly termed digitally reconstructed radiographs (DRR), have been generated in radiotherapy planning for many years.^35^ Maruyama and Yamamoto (2007) used a simplified DRR algorithm in their training simulator. However, their method did not model a divergent radiation beam emanating from a focal point. The resultant parallel rays could not provide the magnification and distortion associated with real radiographs; their simulation was not geometrically accurate for any practical source image distance (SID).^36^ Figure 2 shows the results of the magnification and distortion inherent in projection radiography of the hip as modelled by the computer simulation under investigation in this paper. If these processes are not accurately rendered, then any learning potential must be called into question.

Much previously published research describing DRR algorithms has focused on the accuracy of the transport equation (physics) or the speed of calculation (computer science) rather than the geometric accuracy of the resultant image.^32,37,38^ Nilsson et al. (2004) did set out to geometrically validate an intraoral dental radiography simulator.^39^ Measurements from resultant DRR’s were in close agreement with the expected theoretical length. However, this left the possibility of calculation error and systematic error, as they did not empirically test this agreement by using CT data from a manufactured phantom.

## Methods

This experiment compared the resultant radiographs of a skull phantom with the DRR simulated from 3D data acquired from the same physical phantom. A skull phantom was used, not because skull radiography is commonplace, but because it was judged to contain recognisable bony landmarks that have previously been used for measurement studies in cephalometric analysis,^40,41^ and its sphere shape provided widely spaced landmarks across three perpendicular planes. The radiographs were created using the same acquisition angles and centring points in both the computer simulation and the real X-ray room. Reproducible measurements, sensitive to angulations in all three anatomical planes, were established. These were then measured on both the DRRs and real radiographs. Agreement was assessed using Bland-Altman limits of agreement and Intraclass Correlation Coefficient (ICC) statistics.

It should be noted that the DRRs used for this work are those generated by ProjectionVR^TM^ and therefore only this specific DRR simulation (ProjectionVR^TM^, Shaderware) can be validated from the results. However, this method has wider application and could be used to validate other similar DRR simulations.

### The patient model

The physical patient model was an anthropomorphic skull phantom (3M, Maplewood, MN, USA). The real radiographs were acquired directly from this phantom using projection radiography techniques.

This same patient model was then digitised. The phantom was scanned using a Somatom Sensation 16 slice CT (Siemens Healthcare, Erlangen, Germany). A 184 × 512×512 array of 16 bit voxels was acquired. The pixel size of each tomographic slice was 0.46 mm, the slice thickness was 1 mm and the slice spacing was 1 mm. This data was acquired in collaboration with the company specifically for this research project.

The commercial simulation (ProjectionVR^TM^, Shaderware) is claimed to be ‘fast and capable of running on a wide range of computer hardware’.^17^ There are computational speed advantages to symmetrical arrays when programming directly in video memory using the graphics GPU.^42^ One technique to increase computational efficiency is to resample 16 bit CT data to 8 bit and pack it into a 256 × 256 × 256 array in the simulator (Private communication). Both of these decisions potentially limit the image quality of the final DRRs.

### The process of DRR construction

A fourth Generation Haswell^43^ i5 processor with embedded Intel HD 4600 Graphics was used to run ProjectionVR^TM^. ProjectionVR^TM^ calculates the DRRs from the digitised patient model using a ray casting approach. This process was applied to any data provided to it in the specified proprietary array format, independent of the content of that data.

### The isocentric (Dulac) radiographic projection technique

**Figure 3.**
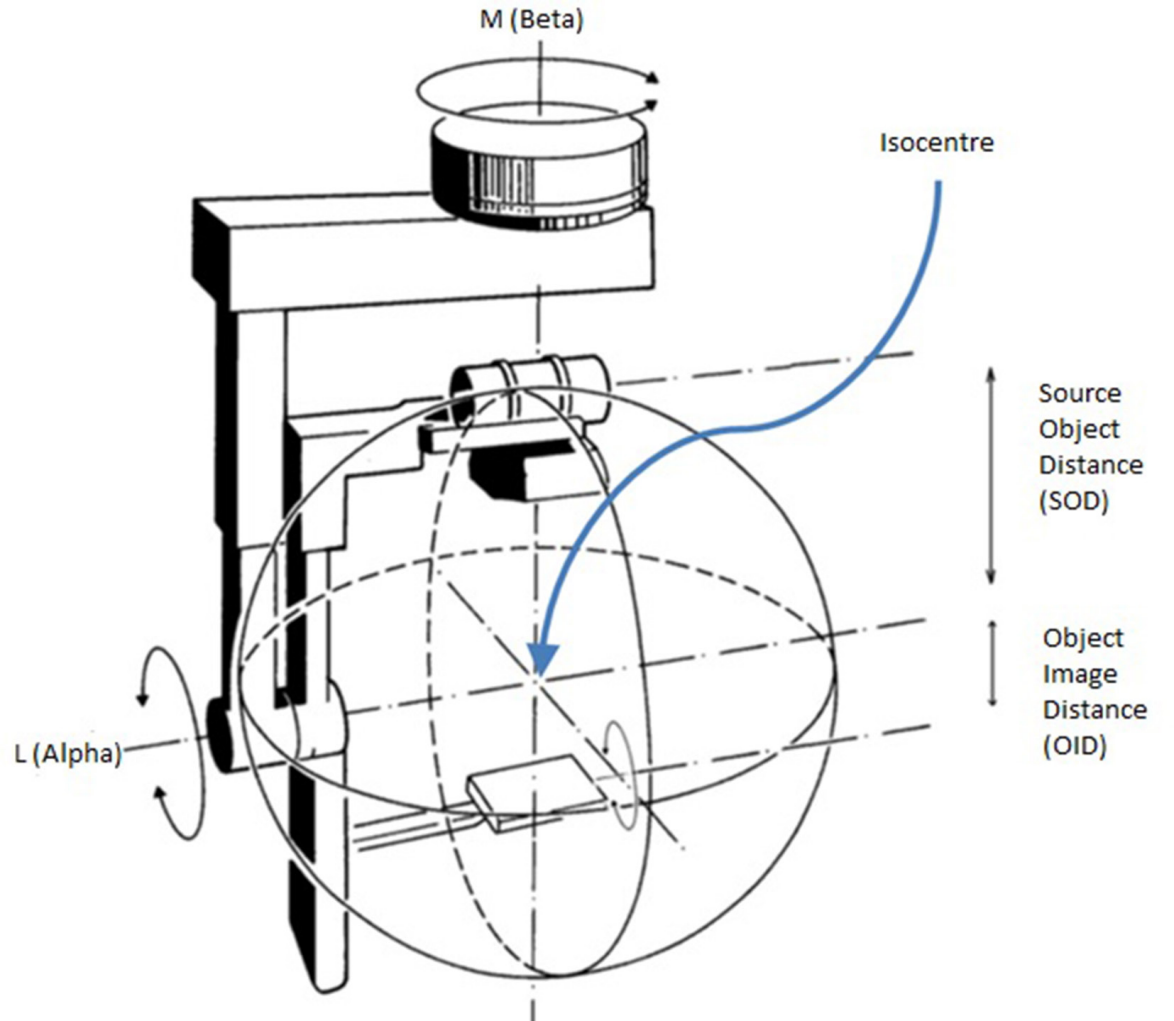
Diagram illustrating the working principles of the Dulac system used to obtain and describe projections acquired.

Isocentric radiographic projection technique, following the Dulac method, has been superseded in the radiography department as skull radiography has given way to CT and MRI as the first-line investigation of head injury and neurological symptoms. However, it was chosen for this validation study as it offers repeatability and accurate notation.^44,45^ The key concept of this system is that an adopted ‘base set up’, usually derived from the anatomical position and the equipment position with respect to this, forms the ‘zero point’. Any projection can then be defined and communicated with respect to this ‘zero point’ by using two modifying axis angles and two table displacements (as long as receptor orientation and angulation are assumed fixed). Reported angles increase from the base in a clockwise direction (Figure 3).

The real radiographs were taken using a Pendo Diagnost isocentric skull unit (Philips Medical Systems). The existing commercial simulation (ProjectionVR^TM^) does not model isocentric equipment. Therefore, a research interface was written by the company to allow input using this system. It reported any change from the base reference setup in displacement of the isocentre, and measurements of degree (0–359), in both the ‘L’ (α) and ‘M’ (β) axes.^44^ All aspects of the ProjectionVR^TM^ DRR algorithm otherwise remained unchanged.

### The radiographic base setup used throughout the study

**Figure 4.**
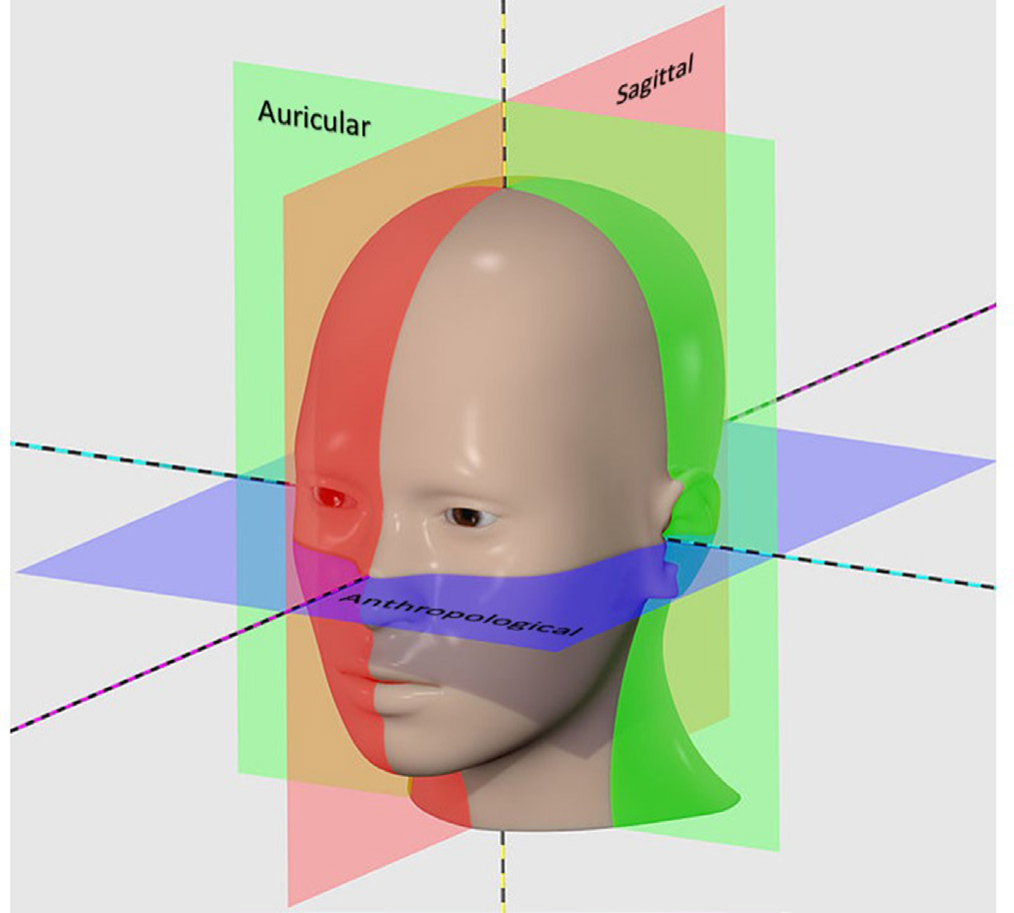
(a) Anthropological (axial, horizontal) plane; (b) Auricular (coronal, frontal) plane; (c) Median sagittal plane.

For both the real radiographs and DRRs, the SID was set to 100 cm throughout. The base reference setup, from which all projections were defined, is described as follows: The central ray (CR) was perpendicular to the coronal plane and parallel to both the median sagittal plane (MSP) and the axial plane of the patient model. Specifically, the axial plane was defined as the anthropological plane (also known as the Frankfurt plane, and identified using Reid’s baseline); cutting through the superior border of both left and right external auditory meatus (EAM) and the infraorbital margins.^44,45^ Both the median sagittal and coronal planes were referenced from this. The coronal plane used was the auricular plane; cutting through the centre of both EAM and perpendicular to the anthropological plane. The MSP was defined as in the midline, perpendicular to both the anthropological and auricular planes (Figure 4).

The CR was positioned to pass through a fixed point at the confluence of these planes termed the isocentre. Any displacement of this point was reported (in mm) as either lower (-ve)/raise (+ve) or cranial (-ve)/caudal (+ve) displacement (no lateral movement was allowed). The object image distance was fixed at 25 cm from the isocentre.

### Obtaining validation data

Real radiographs of the phantom and DRRs were achieved by altering the CR using table displacements and alteration of angles ‘L’ and ‘M’ (Table 1). In the base setup, ‘M’ (β) angle was zero; this axis becomes the point about which rotation in the coronal plane is measured. Zero was reported in the ‘L’ (α) angle when the CR was entering the posterior aspect of the phantom perpendicular to the coronal plane. 90° was recorded in the ‘M’ (β) and ‘L’ (α) angles when the CR was entering the right EAM of the phantom. For each projection, the X-ray film was exposed and checked for optimal optical density and contrast to allow the required bony points to be located.

**Table 1.** Descriptions of the radiographic projections

Projection Name	Left Lateral (Lat)	Pineal (OF 15)	Optic Foramina (FO)
Reference	Kimber (1983)	Kimber (1983)	Maruyama and Yamamoto (2007)*
L (α) angle (degrees)	90	345	145
M (β) angle (degrees)	90	0	125
Table /C displacement (mm)	−40	−40	−40
Table H displacement (mm)	0	0	0
Point of entry of central ray (centring point)	‘40 mm superior to the Rt Porion’	‘2 cm above the external occipital protuberance in the MSP’	‘through the centre of the Rt Orbit’
Exit of central ray (exit point)	‘40 mm superior to the Lt Porion’	‘4 cm above the glabella in the MSP’	‘7.5 cm above and 7.5 cm behind the Lt EAM’
			*reversed

**Figure 5.**
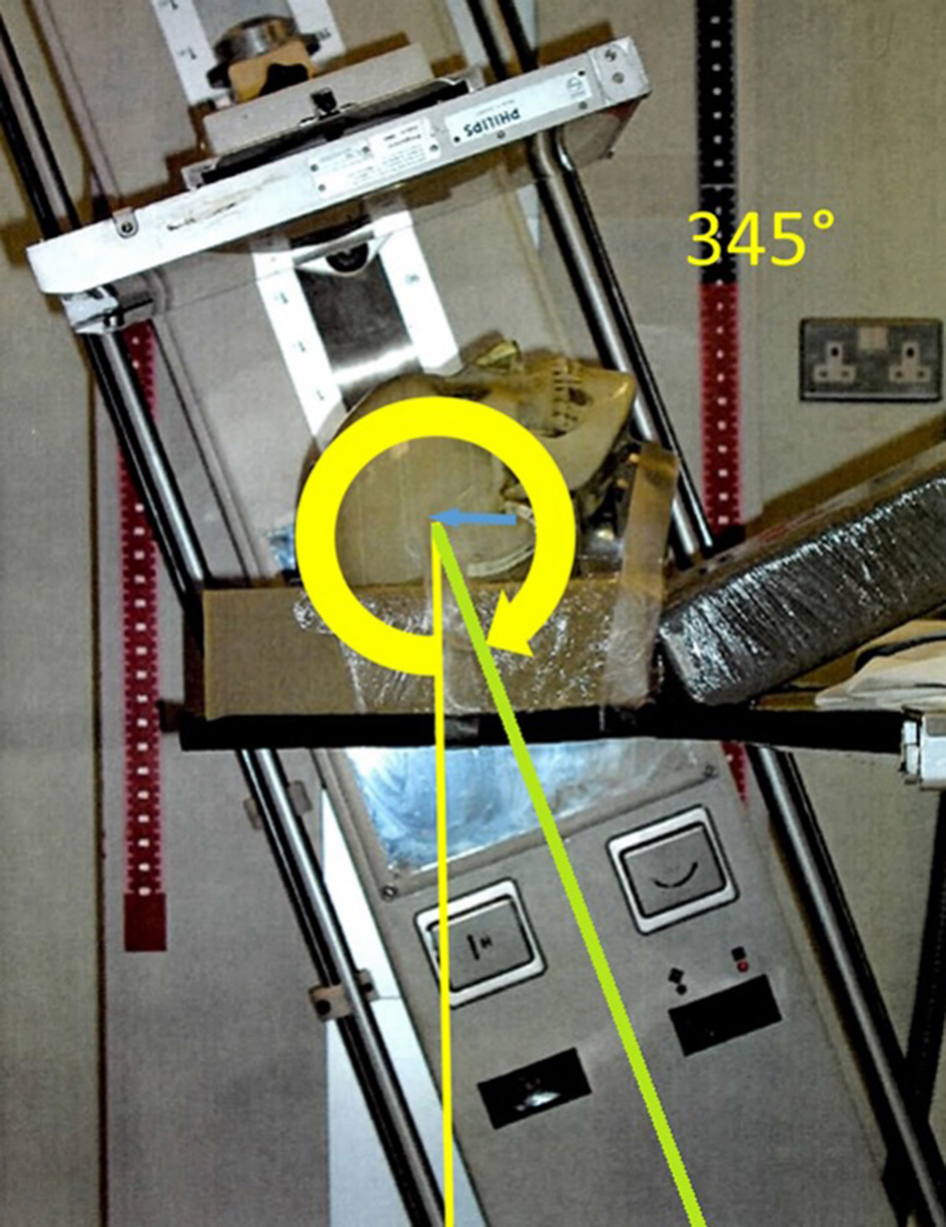
Anthropomorphic phantom in position (Base-40mm) for Pineal Projection (*L* = 345, *M* = 0), CR in green.

Two standard radiographic projections, Occipito-Frontal (Pineal) and Left Lateral, were chosen to provide bony landmarks to validate rotation and distortion in all three anatomical planes.^44,45^ Figure 5 shows the phantom positioned to achieve the pineal projection using the Pendo Diagnost with the CR identified in green. ‘L’ (α) was set to 345 degrees and the isocentre displaced 40 mm in the cranial direction. A third projection was modified from Maruyama and Yamamoto (2007)^36^ to ensure the direction of the CR was noticeably different in the three projections (occipito-frontal, lateral, fronto-occipital). The real radiograph obtained in the ‘Optic Foramina’ projection was then scanned to a digital file for reproduction alongside the DRR.

Once the real radiographs had been obtained, these same coordinates were used as input for the simulation, and the three corresponding DRRs generated. The DRR resolution calculated was 1024×1024 (eight bit); no image quality benefit was seen in higher resolutions. The simulator allowed window width and level to be modified to help locate bony landmarks.

### Test procedure

**Figure 6.**
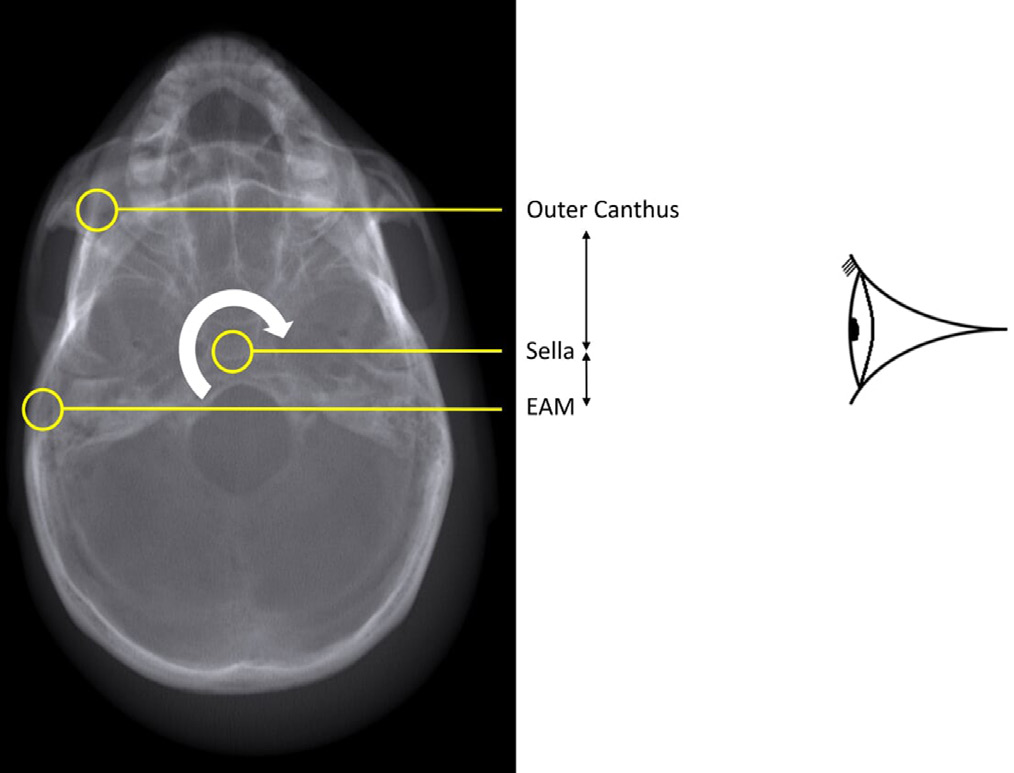
A DRR of the anthropomorphic phantom parallel to the median sagittal plane showing bony points measured in the Occipito-Frontal 15 degrees projection.

**Figure 7.**
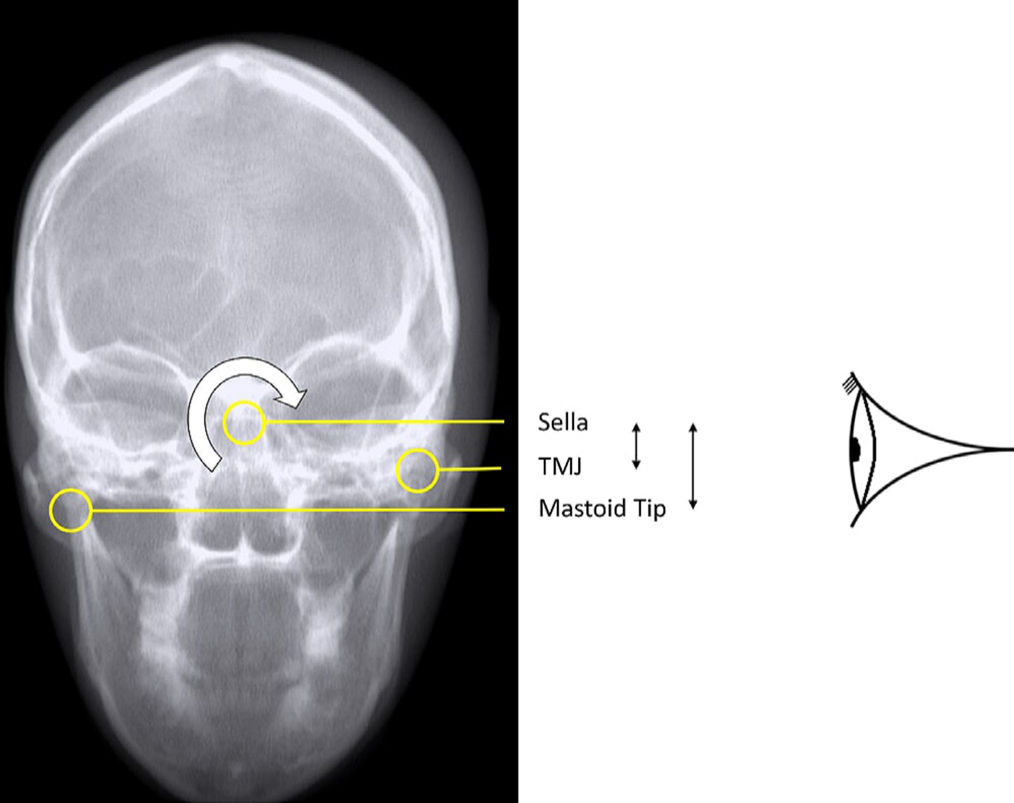
A DRR of the anthropomorphic phantom parallel to the auricular plane (Coronal) showing bony points measured in the lateral projection.

**Figure 8.**
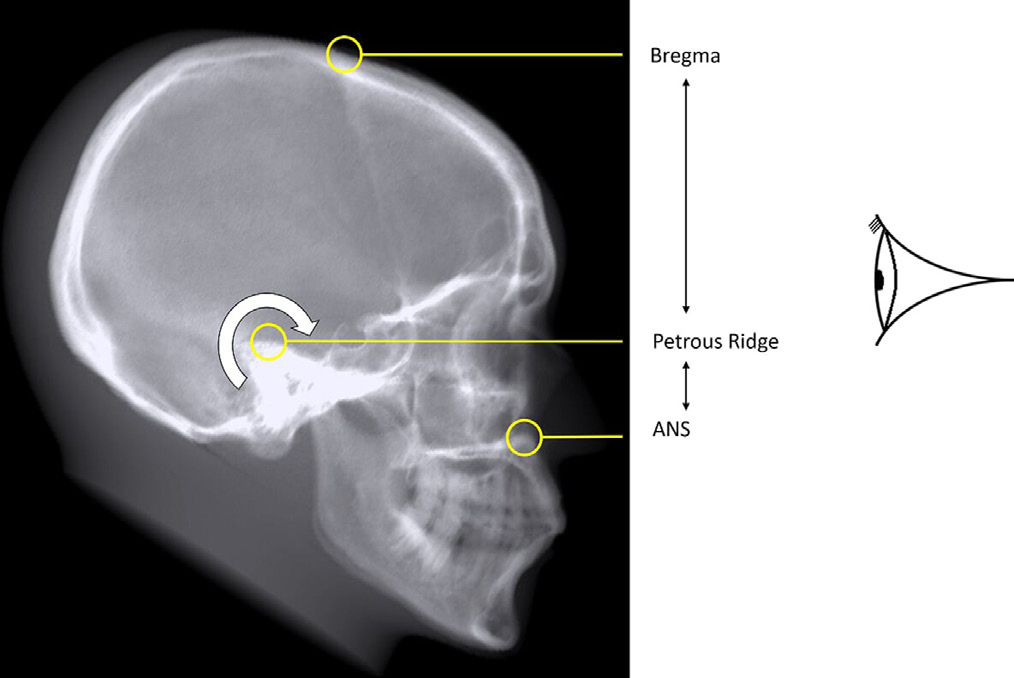
A DRR of the anthropomorphic phantom parallel to the anthropological plane (Axial) showing bony points measured in the lateral projection.

Comparisons were made of the DRRs and the real radiographs using empirical measurements between various standard bony landmarks.^46^ Measurements between points were specifically chosen to have the most sensitivity to rotation in each of three planes; axial, coronal and sagittal. The general principle used was to select a projection where the receptor plane was approximately perpendicular to the plane of measurement and to ensure the measurement involved three widely spaced bony landmarks on the plane (Table 2 and Figures 6–8). For example, any rotation or distortion of projection in the sagittal plane would be seen by a difference in the measured distance between the Bregma, Petrous Ridge and Anterior Nasal Spine (ANS) on the Pineal Radiograph. If the real radiograph and DRR measurements agreed across all planes, then geometric validation had been achieved.

**Table 2.** Bony points, measurements and planes

Bony points	Plane tested	Projection measured
ANS^a^ – Petrous – Bregma	Sagittal	Pineal (OF15)
Rt TMJ^b^ – Sella – Lt TMJ	Coronal	Lt Lateral
Rt Outer Canthus – Sella – Rt EAM^c^	Axial	Lt Lateral

a Anterior Nasal Spine

b Tempero-Mandibular Joint

c External Auditory Meatus

The real radiographs were displayed on 24 × 30 cm X-ray film backlit from an illuminator and the DRRs displayed on a Philips PHL BDM4065 40” 4K LCD monitor capable of presenting at 1:1 scale. Both the radiograph illuminator and the screen were laid flat to facilitate accurate use of a clear plastic ruler; a sheet of 2 mm thick clear acrylic was used to cover the computer screen/radiograph. This is a common method used by orthodontists to monitor patient treatment^47^ and was the method used by Malkoc et al. (2005) to determine the effects of head rotation on cephalometric radiographs.^40^ All measurements were undertaken in a darkened room by four independent observers. The four observers were three radiographers between 10 and 20 years of experience in clinical practice and education and an experienced medical physicist. The measures of point-to-point distances between the relevant bony landmarks were recorded.

### Data analysis

Interobserver agreement was assessed using ICC estimates and their 95% confident intervals. They were calculated using SPSS statistical package v. 24 (SPSS Inc, Chicago, IL) based on a mean-rating (*k* = 4), absolute-agreement and 2-way mixed-effects model. Any value above 0.9 infers excellent agreement.^48^ Analysis of agreement can also be accomplished using graphical techniques and simple calculations, as described by Bland and Atlman.^49^ The final analysis of observations would be by this method if the ICC was considered ‘excellent’.

## Results

Table 3 provides six measured bony point-to-point distances, where the landmarks were widely spaced across three anatomical planes, from each of the four observers from real and DRR images. Variation of between 3 and 17% (1–6 mm) was recorded between the four observers. However, the ICC was 0.999 (95% CI: 0.996–1.000). This is considered an ‘excellent’ agreement, allowing the mean of all observers to be taken forward for the final analysis.

**Table 3.** Measurements taken from images by observers

Measurement	Observer 1		Observer 2		Observer 3		Observer 4	
	Real (mm)	DRR (mm)	Real (mm)	DRR (mm)	Real (mm)	DRR (mm)	Real (mm)	DRR (mm)
ANS-Petrous	47	47	48	50	51	47	47	45
Petrous-Bregma	119	117	116	118	110	114	119	116
Lt. TMJ-Sella	26	27	25	28	28	28	27	26
Sella-Rt. Mastoid	66	62	66	64	65	67	68	65
Lt. Outer Canthus-Sella	38	39	37	34	37	34	37	33
Sella-Lt. EAM	32	32	38	33	33	32	35	33

**Figure 9.**
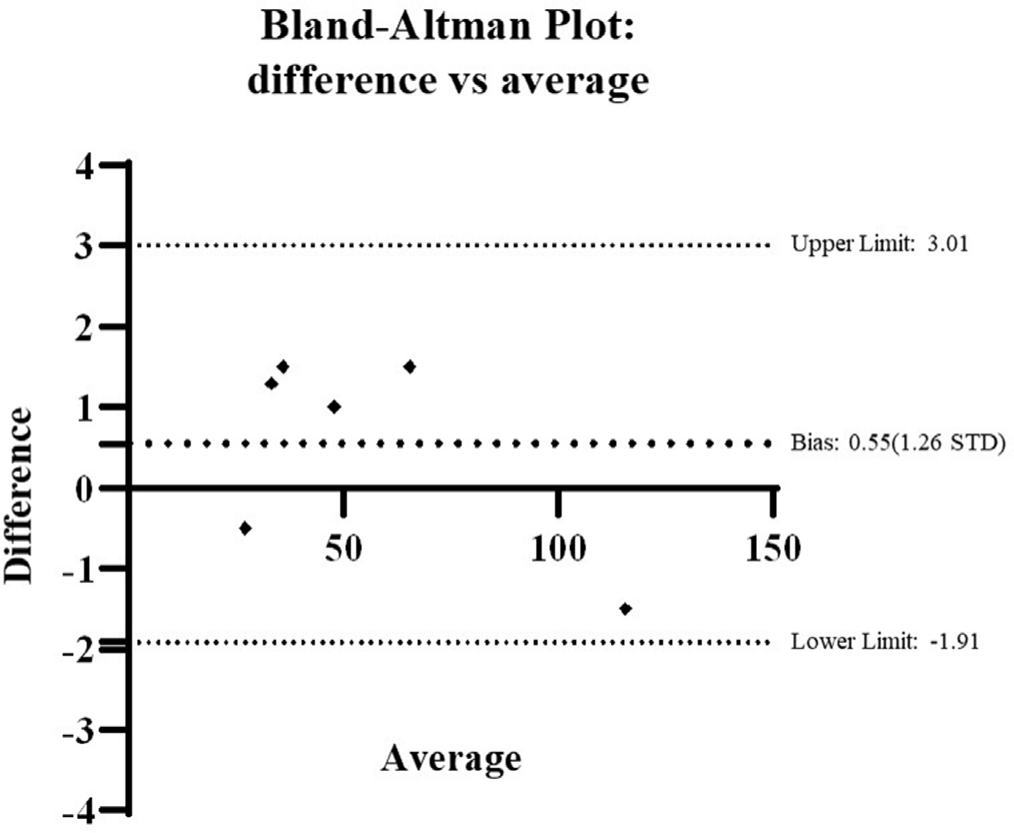
Bland-Altman plot presenting limits of agreement and bias for observer means.

Table 4 provides the average and standard deviation of these six mean measures taken from both real and DRR images, and the difference of the means between real and DRR images. This analysis of the mean observations shows that the measurement from the DRR matches the real radiograph +1.5 mm/−1.5 mm. The Bland-Altman bias was 0.55 (1.26 STD), with 95% limits of Agreement 3.01 to −1.91 (Figure 9).

**Table 4.** Mean, standard deviation and difference estimates for each plane and measurement

Validating plane	Measurement subject	Measurements from real (mm)		Measurements from DRR (mm)		Difference between means (mm)
		Mean	STD	Mean	STD	
sagittal	ANS-Petrous	48.3	1.9	47.3	2.1	1.0
sagittal	Petrous-Bregma	114.8	3.8	116.3	1.7	−1.5
coronal	Lt. TMJ-Sella	26.8	1.5	27.3	1.0	−0.5
coronal	Sella-Rt. Mastoid	66.0	0.8	64.5	2.1	1.5
axial	Lt. O. Canthus-Sella	36.5	1.7	35.0	2.7	1.5
axial	Sella-Lt. EAM	33.8	2.9	32.5	0.6	1.3

**Figure 10.**
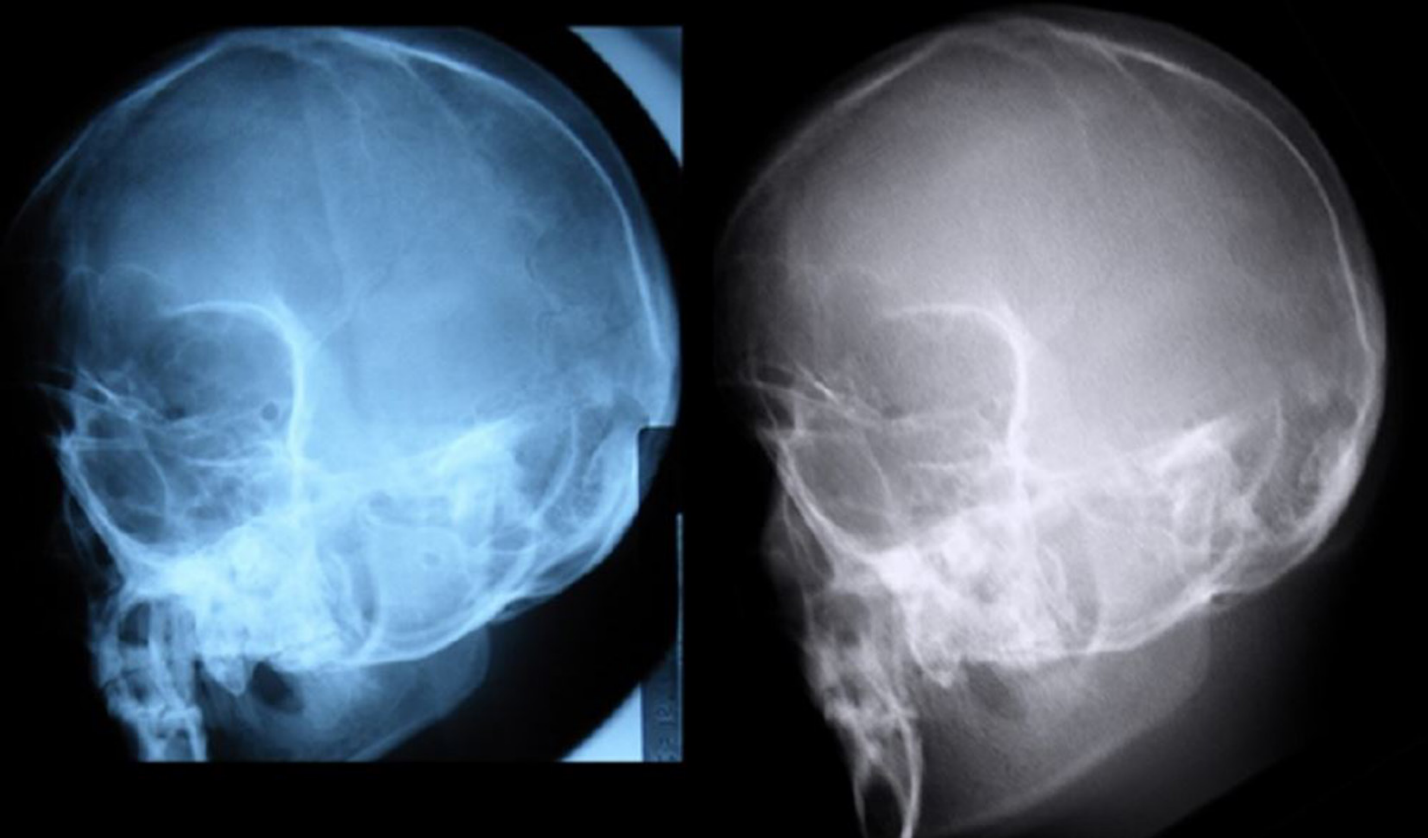
(a) real and (b) virtual radiograph Optic Foramina projections compared.

A qualitative comparison between real radiograph and DRR is provided (Figure 10).

## Discussion

Practical classes provided for student radiographers to gain feedback on their radiographic methods require accurate representation of radiographic projections, either from irradiating plastic phantoms using real X-ray equipment or computer simulations such as ProjectionVR^TM^. A method of validating a computer simulation of radiographic image generation has been outlined. This methodology could be used on other commercially available simulations.

Using this methodology, comparison between measurements taken from real radiographs and the corresponding DRRs generated by ProjectionVR^TM^ demonstrate an agreement of measures between bony points ± 1.5 mm, which is within the bounds of normal intra observer variation in clinical cephalometric measures.^50^ This constitutes evidence of geometric validation of the DRR algorithm underpinning this widely used commercial diagnostic radiography simulator. Objectively accurate DRRs, directly comparable to real radiographs, have been generated from a phantom in two differing projections; the simulation accurately mimicked distortion in all anatomical planes due to projection and magnification (Figure 10).

There are limitations to this work. The observers recorded only one measurement of each point-to-point distance where it is good practice to repeat the measurement three times and take an average (to guard against aberrant data points); repeated measurement should be adopted in any future work. The real radiographs were acquired using film-screen technology. This had the advantage of removing any possibility that distortion and magnification were derived from issues with display technologies, but the inability to change the greyscale presentation of the radiograph might have led to difficulty in identifying the bony landmarks for point-to-point measurement.

The data used in the commercial software offered for sale is derived from cadaver data, not this research data as tested, and the interface was modified to allow specific control and reporting; this does affect generalisability of the work. The interface changes will not affect the calculation of the DRRs in any way, and the cadaver data is acquired, stored and processed in the same way as the research data has been. It is therefore expected that any DRRs of other body parts calculated using the commercial product will be no different in accuracy.

Use of Dulac’s isocentric technique, while obsolete in clinical practice, was useful in describing accurately projection geometry for research purposes. However, it introduced a possible limitation to this work in that no radiographs were taken with a receptor that was not perpendicular to the CR.

One interesting and unexpected outcome was demonstrated by DRRs generated from the physical phantom in the cardinal planes (Figures 6–8). These show that the skull embedded in the anthropological phantom is misaligned with the plastic surface markings; there is a considerable difference in the plastic thickness from front-to-back and side-to-side. If the phantom had not been transparent, allowing the sighting of bony landmarks through the plastic surface, its use in radiography education might lead to a negative training effect.

## Conclusion

Geometric validation of this particular commercial computer simulation algorithm has been evidenced using an experimental data set from an anthropomorphic phantom. These results are reassuring since thousands of students have used a computer simulation based on the same algorithm in their training over many years across the world. Any danger of a negative training effect due to incorrect simulation of magnification or distortion can be discounted.

This work does not constitute full validation. Other projections of different anatomical areas would provide more data. There are also good reasons to conduct future research to consider the simulations in-built radiometric and dosimetric models. These control the greyscale presentation and dose estimation feedback given by this simulator. To this end, the developers have included an 11-step aluminium step-wedge and a known thickness of Polymethyl methacrylate (PMMA) as selectable objects for irradiation. These will allow researchers to conduct future validation studies.

The anthropomorphic phantom used in this study proved to have the bony skull misaligned with the plastic moulded surface markings, such as nasion, glabella and EAM. This may be unusual and a finding limited to this one phantom. However, future work may be conducted to discover if this is a common fault; there is the potential for opaque plastic physical phantoms to be responsible for negative feedback to students.

Any simulator intended to be used as a training or assessment tool will need sufficient evidence of transfer of training from time in simulation to real measured improvement in radiographic skills. Should this evidence be forthcoming, there are ethical, financial and educational arguments in favour of computer simulation augmenting phantom simulation in the energised lab and time in clinical placement.
